# Supplementary figures and images for: Hepatoprotective Effects of Sweet Cherry Extracts (cv. Saco)
Source: Foods. 2021 Oct 29;10(11):2623. doi: 10.3390/foods10112623 (PMC8621173; doi:10.3390/foods10112623)

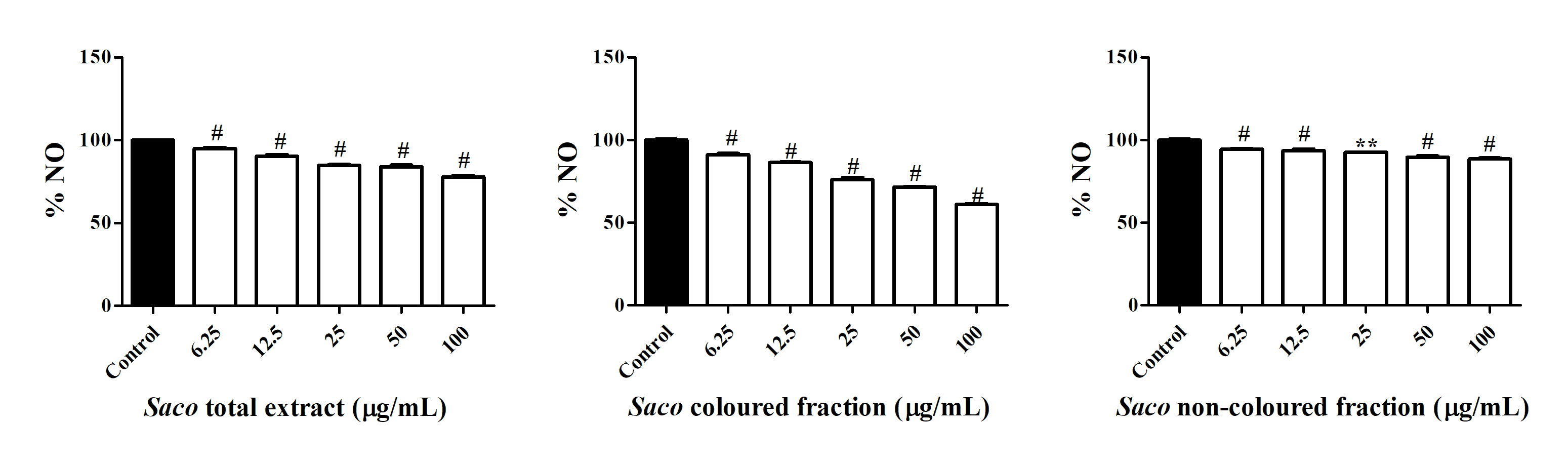

Supplement: Supplementary file 1 [file foods-10-02623-s001.zip › Figure S2.tif]
